# Predictors of discordant MRSA nares and respiratory cultures in patients with pneumonia

**DOI:** 10.1093/jacamr/dlac095

**Published:** 2022-09-30

**Authors:** Nicholas P Palisano, Christina F Yen, Nicholas J Mercuro

**Affiliations:** Department of Pharmacy, Beth Israel Deaconess Medical Center, 330 Brookline Ave, Boston, MA 02215, USA; Division of Infectious Diseases and Geographic Medicine, Department of Internal Medicine, University of Texas Southwestern Medical Center, 5323 Harry Hines Blvd, Dallas, TX 75201, USA; Department of Pharmacy, Maine Medical Center, 22 Bramhall St., Portland, ME 04102, USA

Current guidelines for pneumonia management recommend empirical therapy for MDR organisms, including MRSA, in high-risk patients.^1,2^ MRSA nares swabs facilitate de-escalation of empirical antimicrobials, with a reported negative predictive value (NPV) of approximately 96%–99% for MRSA pneumonia.^3–6^ However, concerns for swab and respiratory culture discordance remain, and there are a few known risk factors associated with discordant results.^4,6^ We conducted a single-centre, retrospective, case–control study to evaluate predictors of having a discordant result (negative MRSA nares swab with a positive MRSA respiratory culture) in patients diagnosed with pneumonia. Chi-squared and Fisher’s exact test were used to compare categorical variables and Mann–Whitney *U*-test was used for continuous variables. Associations with discordant results were described with OR and 95% CI.

Between January 2014 and December 2020, the frequency of discordant cases among 1438 hospitalized adults was 0.28%. Forty-six cases of discordance were matched to 138 controls (negative MRSA nares swab and a respiratory culture without MRSA growth) based on the year and location (ICU versus wards) of swab collection (Table 1). The mean (SD) age was 67.2 (15.2) years with 117 (63.6%) males. Overall, 115 (62.5%) patients were mechanically ventilated for a median duration of 1 day (IQR 0–3) prior to MRSA swab collection and 124 (67.4%) were in the ICU at the time of collection. The median time from admission to MRSA nares collection was 1.5 days (IQR 0–5). The time between MRSA nares and respiratory culture collection was longer in the case group [2 (IQR 0–8) versus 0 (IQR 0–1) days, *P < *0.001]. MRSA swabs were collected prior to or on the same day as respiratory cultures in 145 (78.8%) subjects. Three subjects (1.6%) had a respiratory sample obtained more than 14 days after the nares swab. Hospital length of stay was longer in the discordant case group compared with the control group [26 (IQR 13–42) versus 16 (IQR 10–24) days, *P < *0.001].

**Table 1.** Baseline characteristics and univariate analysis of potential risk factors for having negative MRSA nares swabs with a positive MRSA respiratory culture result in patients diagnosed with pneumonia

	Cases (*n *= 46)	Controls (*n *= 138)	*P* value	Unadjusted OR (95% CI)
Age, years, mean (SD)	64.5 (17.7)	68.1 (14.2)	0.51	—
BMI, kg/m^2^, mean (SD)	29.6 (9.3)	28.3 (7.4)	0.58	—
Race, *n* (%)			0.68	—
Caucasian	28 (60.9)	85 (61.6)		
African American	5 (10.9)	15 (10.9)		
Other/not reported	13 (28.2)	38 (27.5)		
Charlson comorbidity index, median (IQR)	4 (2–6)	5 (3–7)	0.23	—
Diabetes mellitus, *n* (%)	19 (41.3)	47 (34.1)	0.38	1.36 (0.69–2.70)
Renal replacement therapy, *n* (%)	4 (8.7)	6 (4.3)	0.27	2.10 (0.56–7.78)
Immunocompromised^a^, *n* (%)	2 (4.3)	24 (17.4)	0.028	0.22 (0.05–0.95)
Chronic lung disease, *n* (%)	12 (26.1)	27 (19.6)	0.35	1.45 (0.66–3.17)
Surgery within prior 90 days, *n* (%)	10 (21.7)	21 (15.2)	0.31	1.55 (0.67–3.59)
Surgery during hospitalization, *n* (%)	14 (30.4)	41 (29.7)	0.93	1.04 (0.50–2.14)
Length of stay, days, median (IQR)	26 (13–42)	16 (10–24)	<0.001	—
Central venous catheter, *n* (%)	26 (57)	61 (44)	0.15	1.64 (0.84–3.22)
Vasopressor support, *n* (%)	21 (45.7)	60 (43.5)	0.80	1.09 (0.56–2.14)
Mechanically ventilated prior to MRSA nares, *n* (%)	30 (65.2)	85 (61.6)	0.66	1.17 (0.58–2.35)
Duration of mechanical ventilation, days, median (IQR)	1 (0–3)	1 (0–3)	0.72	—
Time to MRSA nares, days, median (IQR)	1 (0–3)	2 (0–5)	0.27	—
Time between admission and MRSA nares ≥7 days, *n* (%)	7 (15.2)	21 (15.2)	1.00	1.00 (0.40–2.53)
Time between MRSA nares and respiratory culture ≥7 days, *n* (%)	14 (30.4)	3 (2.2)	<0.001	19.69 (5.34–72.61)
Vancomycin exposure prior to nares collection, *n* (%)	13 (28.3)	62 (44.9)	0.046	0.48 (0.23–1.00)
Prior history of MRSA infection or colonization, *n* (%)	4 (8.7)	1 (0.7)	0.014	13.05 (1.42–119.94)

a HIV/AIDS, solid organ transplant on immunosuppressants, recent stem-cell or bone-marrow transplant, active chemotherapy, immunosuppressive medications.

The NPV of the MRSA nares swab for MRSA pneumonia was 96.8%. Per local antibiogram data, the prevalence of MRSA among all *Staphylococcus aureus* clinical cultures ranged from 31% to 37% during the study period. The median duration of vancomycin after negative MRSA nares results was longer in cases than controls [4.5 (IQR 3.0–8.5) versus 1 (IQR 1–2) days, *P < *0.001]. A history of MRSA infection [OR 13.05 (95% CI 1.42–119.94)] and time between swab and respiratory culture collection of ≥7 days (OR 19.69 [95% CI 5.34–72.61]) were predictors associated with test discordance. Immunocompromised status was associated with test concordance [OR 0.22 (95% CI 0.05–0.95)]. There was no difference between respiratory specimen collection between groups, with bronchoalveolar lavage occurring in 30.4% and 20.3% of cases and controls, respectively (*P *= 0.15). Frequency of acute kidney injury (AKI) [defined as an increase in serum creatinine (SCr) ≥0.5 mg/dL or a 50% increase from SCr on the day of vancomycin initiation^7^] was similar between groups (12.5% versus 21.1%, *P = *0.67). There was no difference in hospital mortality between cases and controls (17.4% versus 12.3%, *P = *0.39).

De-escalation of anti-MRSA therapies for respiratory infections following negative MRSA nares screening is an accepted practice in antimicrobial stewardship.^1,2^ However, patients with pneumonia and negative MRSA nares swabs are often continued on potentially harmful anti-MRSA therapies.^4^ In our study, 13 patients (7.1%) with concordant negative results being treated with anti-MRSA therapy exclusively for pneumonia were continued on therapy.

The acceptable time between MRSA nares screening and respiratory culture collection remains an area of investigation. A recent study reported an NPV of 95.5% when the time between MRSA nares and respiratory sample collection was 14 days.^6^ We found that history of MRSA infection and time of ≥7 days between swab and respiratory culture were associated with discordance. However, the low event rate limits the ability to control for other confounders and interpretation of findings should be approached carefully based on the nature of the retrospective design. We applied a 7 day cut-off point to focus on discordance within the same hospitalization, and few subjects (1.6%) had a respiratory sample obtained >14 days after the nares swab. While these duration cut-off points have been identified in the literature, the NPV remains high even beyond 7 and 14 days.^6,8^ Mallidi *et al.*^8^ calculated an NPV >98% in critically ill patients using a duration between swab and respiratory sample collection of up to 60 days. Ultimately, extrapolation to other institutions is dependent on local prevalence and testing practices.

In conclusion, negative MRSA nares testing with a subsequent positive respiratory culture is a rare occurrence, supporting de-escalation of anti-MRSA therapies for patients with pneumonia. In patients with suspected pneumonia who have a history of MRSA infection or an MRSA swab collected ≥7 days prior, a high-quality respiratory sample should be pursued for diagnostic purposes as opposed to repeating an MRSA swab.
